# Characterizing deterioration of milk through bioimpedance spectroscopy

**DOI:** 10.2142/biophysico.bppb-v22.0016

**Published:** 2025-08-02

**Authors:** Abhishek Mallick, Bishal Paul, Anirudhha Roy, Arijit Roy

**Affiliations:** 1 Department of Electronics, West Bengal State University, Kolkata, Barasat 700126, India; 2 JIS School of Medical Science and Research, West Bengal, Howrah 711112, India; 3 Indian Institute of Science Education and Research, Kolkata, Mohanpur 741246, India

**Keywords:** adulteration, ANOVA, bioimpedance, Cole parameters, milk

## Abstract

Quality assessment and characterization of liquid milk by means of electronic sensor remains an intensive area of research. In this work, cylinder-in-cylinder type sample holder is fabricated to measure the bioimpedance of milk as a function of frequency. Experiments on bioimpedance spectroscopy were conducted during adulteration (at control temperature, 23°C) of branded (pasteurized) milk as well as raw milk. Cole equivalent circuit is considered as a characterizing model for milk. Cole parameters were extracted from the experimental data. From Cole parameters, relaxation-time was estimated and state of the milk sample has been expressed in terms of relaxation-time. Analysis of variance was performed on relaxation-time of the samples to gain statistical significance. Our method is capable to discriminate liquid milk from different commercial brands. It was found that the relaxation-time decreases monotonically with the progression of adulteration time for all kinds of milk considered in this study. From the changes in relaxation-time, the adulteration was found to be significant in the first three hours. Hence it is not advisable to consume milk after two hours of adulteration if kept at 23°C. Dominant biochemical pathways responsible for adulteration of milk are also presented.

## Significance

The well-established standard Cole equivalent circuit is considered for the bioimpedance spectroscopy (BIS) study of milk. All Cole parameters are experimentally estimated to characterize the state of the milk undergoing adulteration process. Relative changes in relaxation-time is considered as the monitoring parameter for adulteration. Instead of single measurement, multiple measurement is conducted and ANOVA is incorporated in the BIS study to gain statistical relevance. A simple low-cost sample holder is fabricated to study BIS of liquid sample. As an alternative to traditional pathological test, shelf-life of milk is estimated by electrical test.

## Introduction

Liquid milk remains as an essential source of nourishment for the human body and is consumed worldwide. Milk contains various nutrients including sugars, proteins, fats, minerals etc. and it is a balanced food. The nutritional content of milk depends on the source and the processing methods it is subjected to. Therefore, the exact composition and quality of milk depends on its processing history and hence, varies from brand to brand. Liquid milk requires careful tests because of its natural tendency towards adulteration and rapid degradation [1–5] even when kept at room temperature.

Several studies have reported to predict the shelf-life, degradation, and adulteration of milk [1–11]. Conventional pathological tests on the shelf-life of pasteurized whole milk under refrigerated conditions have been conducted and predictive models for quality loss have been reported [2]. Similar conventional tests have also been used to study the effect of post-pasteurization contamination on the quality of liquid milk [3,4]. In the quality control assessment of liquid milk, alternative test methods are emerging since, conventional pathological tests are labor-intensive, time consuming and expensive.

Methods of electrical characterization of milk are among the various alternatives which are emerging recently [1,5–15]. These characterization methods are BIS, Dielectric Spectroscopy (DS), Electrical Conductivity (EC) etc. In order to assess the quality of milk, BIS study is conducted extensively to quantify the effect of water-content (dilution) and temperature; and alternative (non-standard) equivalent circuits for milk have been presented [5,6]. The presence of pathogenic bacteria (such as *E. coli*), poses a significant threat to milk quality. BIS has been used to monitor bacterial growth over time, and corresponding equivalent circuit for such biological systems have been presented [7]. Additionally, quantitative evaluation of fat content in raw milk has been attempted using BIS method [8]. Characterization of ultra-high-temperature (UHT) pasteurized milk (available at grocery stores and supermarkets in Europe) has been studied using BIS method and classification of milk such as whole, semi-skimmed, fat-free and to distinguish lactose-free milk has been presented [9]. The adulterated concentration, freshness of milk; identification and quantification of various polar and non-polar/ionic adulterants have also been presented by studying the DS and impedimetric measurements of milk [10–12]. Before we proceed further, we would like to mention that adulteration, spoilage and deterioration of milk have been treated as synonyms in this paper.

In the above-mentioned studies, the authors have presented their own equivalent circuits for milk by fitting their experimental data. Thus, a number of equivalent circuits exist, and because of this reason, the model parameters used to express the state of a milk sample cannot be compared or correlated. Further, these studies are based on single measurement method. Multiple measurement and statistical analysis on the measured data have not been considered, which is another disadvantage of these studies. On the other hand, Cole equivalent circuit is a well-established model and has been extensively used in BIS study [16–20]. In fact, Cole equivalent circuit is used for diagnosis of breast cancer [13], human skin [14], fruits and vegetables [18–20] etc.

Consideration of a standard equivalent circuit is extremely important for universal application. Nevertheless, in this study we have considered standard equivalent circuit (the well-known Cole equivalent circuit) and estimated the parameters of interest experimentally with statistical relevance. In this paper, we have presented BIS study of commercial milk of two brands along with raw milk considering Cole equivalent circuit as the standard bioimpedance model. For the measurement of bioimpedance, a special fixture (sample holder) was made for containing the liquid sample. The degradation and/or adulteration of milk with time at control temperature was studied and the physiological sate of the sample is described in terms of Cole parameters. From the Cole parameters, the relaxation-time was computed. Extensive analysis of variance (ANOVA) was performed on the relaxation-time to obtain statistical relevance of our study.

## Cole bioimpedance model

**Figure 1:**
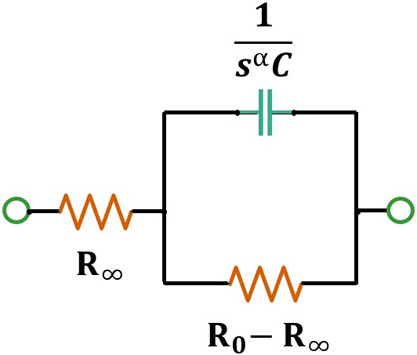
Cole equivalent circuit for biological body [17,18]. Here, s=jω.

Single dispersion Cole equivalent circuit is well accepted model for bioimpedance measurement [17,18,20–25]. The model’s excellent co-relation with experimental findings is the reason of its wide acceptability for BIS study. The single dispersion Cole equivalent circuit for lump biological subject is shown in Figure 1. The equivalent circuit consists of a constant phase element (CPE) comprising of a fractional capacitor (C) with a parallel resistance (R_0_–R_∞_) and a series resistance R_∞_. The CPE maintains the phase invariancy by the dispersion parameter α. In the equivalent circuit, R_0_ is the dc resistance (or low frequency resistance of the sample); R_∞_ is the ac resistance of the same at high frequency.

The impedance of single dispersion equivalent circuit (Cole equivalent circuit) is usually expressed as:

(1) $$Z=R_{\infty }+\frac{\left(R_{0}-R_{\infty }\right)}{1+(j\omega \tau {)}^{\alpha }}$$

where, $j=\sqrt{-1}.$ The symbol τ in Eq. (1) is known as the characteristic time-constant (relaxation-time) of the biological object under consideration and is expressed as:

(2) τ=[(R0−R∞)C]1α

Separation of Z into real and imaginary parts is not straight forward due to the non-integer value of α(0<α≤1), which turns the Eq. (1) into the category of fractional calculus. Nevertheless, analytically it is possible to express the impedance Z in terms of real and imaginary parts. After simplifying, the modulus of Z is expressed as (for derivation, readers are referred to references [17,18]):

(3) $$|Z|=\sqrt{(X{)}^{2}+(Y{)}^{2}}$$

The real and imaginary parts of Z are:

(4) $$X=R_{\infty }+\frac{\left(R_{0}-R_{\infty }\right)\left(1+\omega^{\alpha }\tau^{\alpha }\cos\frac{\alpha \pi }{2}\right)}{{\left(1+\omega^{\alpha }\tau^{\alpha }\cos\frac{\alpha \pi }{2}\right)}^{2}+{{(\omega }^{\alpha }\tau^{\alpha }\sin\frac{\alpha \pi }{2})}^{2}}\;\text{and}$$

(5) $$Y=-\frac{\left(R_{0}-R_{\infty }\right)\left(\omega^{\alpha }\tau^{\alpha }\sin\frac{\alpha \pi }{2}\right)}{{\left(1+\omega^{\alpha }\tau^{\alpha }\cos\frac{\alpha \pi }{2}\right)}^{2}+{{(\omega }^{\alpha }\tau^{\alpha }\sin\frac{\alpha \pi }{2})}^{2}}\;\text{respectively.}$$

Experimentally, |Z| is measured as a function of frequency *f* (=ω2π). The experimental data is then fitted (by non-linear curve fitting method) and values *C* and α are obtained. In the non-linear curve fitting process, R_0_ and R_∞_ are taken as measured impedance at lowest and highest frequency respectively (the procedure of non-linear curve fitting is described in later section of this article). Thus, knowing the values of R_0_, R_∞_, *C* and α, the relaxation-time (τ) is calculated using Eq. (2).

## Fabrication of sample holder and experimental setup

**Figure 2:**
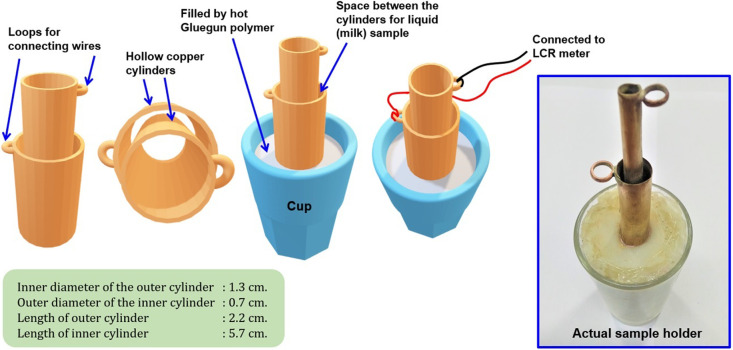
Design and construction of sample holder for liquid sample (milk).

In the study of BIS, two-electrode or four-electrode configurations are commonly used. Each of these configurations have their own advantages and disadvantages. However, in order to obtain electrical access to the liquid milk samples, a two-electrode system was adapted, similar to the methods used in references [1,6,9,12]. In order to measure impedance of a liquid sample, two concentric cylinders system (also known as cylinder-in-cylinder system) was designed. In this kind of sample holder, the gap between the cylinders was filled with the liquid under investigation while the cylinders served as electrodes. A similar technique is adapted in this work (a study on milk with cylinder-in-cylinder can be found in reference [9,11]). We have designed and fabricated a custom sample holder for our study and the construction has been described in the Figure 2.

**Figure 3:**
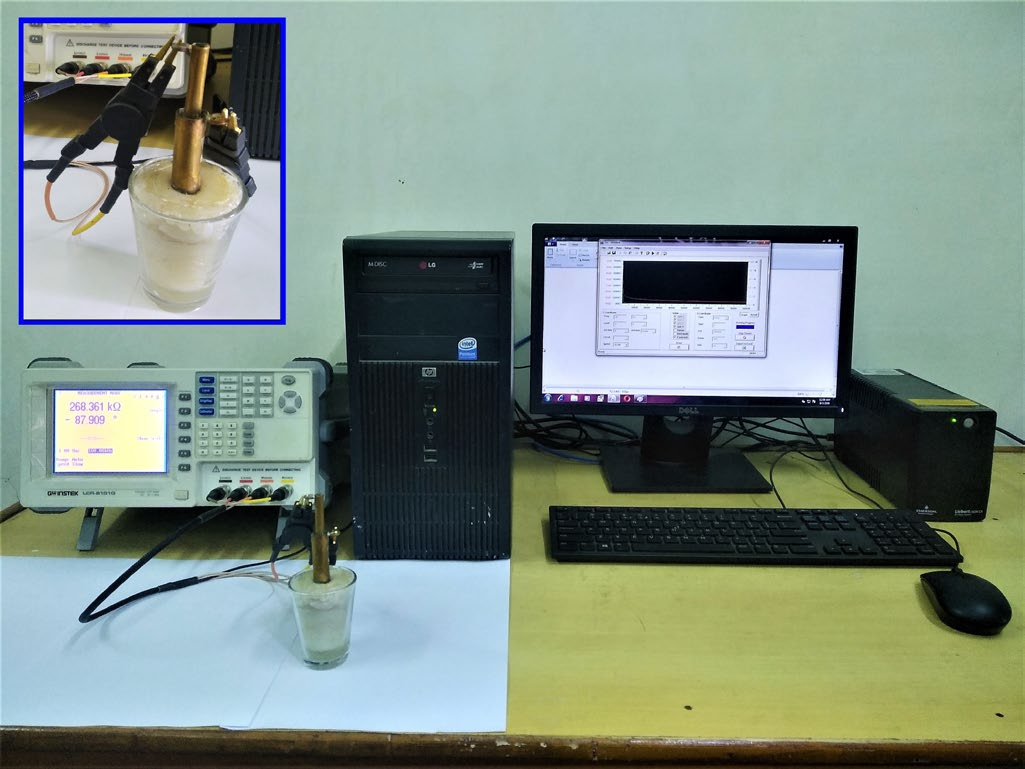
The entire experimental setup. Inset: Sample holder and its electrical connection.

The cylinders of the sample holder were made of thin sheet of copper. The sample holder was fitted in a small transparent glass, at the center by means of glue gun. All the dimensions of various parts of our sample holder were comparable to that of commercial sample holder (commonly used to measure dielectric constant of liquid sample). The sample holder served basically as a capacitive sensor that is used to study BIS of milk [1,6,8,9,11]. A disposable syringe was used to pour the sample (milk) into the gap between the cylinders up to a height of the outer cylinder. The inner and outer cylinder served as two electrodes and were connected to LCR meter. The LCR meter (GWINSTEK LCR8101G) was connected to PC for communication with the LCR meter. The entire experimental setup is shown in Figure 3.

## Sample matrix and bioimpedance measurement

A total of three different types of milk were considered in this study. Pasteurized milk from two different brands (containing 3% fat) as well as raw milk (fat percentage is not known) were considered as samples in our study. The branded milk was purchased from two different shops to check the shop-to-shop variability, if any. The description of these samples is depicted in Table 1.

The impedance of the sample (milk) was measured in the frequency range of 1 Hz to 1 MHz with 399 data points. First 200 data points were taken in the range of 1 Hz to 5 kHz and the next 199 data points were taken in the range of 10 kHz to 1 MHz. The data points were taken at linear increment of frequency. The LCR meter was controlled through a desktop PC, using the graphical user interface of the LCR meter. The experimental data from the LCR meter was communicated to the PC and was stored as a MS Excel file. The Cole parameter R_0_ and R_∞_ are the measured impedance at 1 Hz and 1 MHz respectively. For each set of data, the non-linear curve fitting was performed and relaxation-time (τ) was computed using Eq. (2). It is to be noted here that the Cole parameters, R_0_, R_∞_, and C can only be treated as characteristics of the sample if the sample holder is standardized. On the other hand, the Cole parameters, α and τ, both are fundamental characteristic parameters of a particular sample and is independent of size and shape of the sample holder. This means that the parameters α and τ are independent of the method of measurement. Nevertheless, instead of the actual values of these parameters, the goal of this work is to find the changes in their values (especially in τ) over time during the adulteration process.

The non-linear curve fitting (measured |Z| with the right-hand side of the expression given by Eq. (3)) was performed using software package MS Excel by an iterative method. The goodness-of-fit is measured by R-Squared value. For each set of data, the curve fitting was iterated until the R-Squared value reaches to at least 0.99. The outcome of an iteration was used as the guess parameters for the next iteration. The guess parameters for the first iteration were 0.5 and 0.0001 for the parameters α and C respectively. The relaxation-time, τ was then calculated using values of α and C obtained from the last iteration using Eq. (2). The relaxation time (τ) is monitored over time during the adulteration process. If the change in τ is statistically significant, then the sample is considered to be adulterated.

In order to obtain statistical relevance, each bioimpedance measurement consists of five replica of impedance measurements as a function of frequency. Ideally, the Cole parameters should be identical for a given brand of milk irrespective of their sources. Conversely, this means that the source of milk for a particular brand should not be a factor in the variation of Cole parameters. In order to prove this hypothesis, impedances of all the samples were measured and distinguishability of the sources were checked and it has been proven here that the variation due to ‘Source’ is statistically insignificant (see results and discussion section for details).

**Figure 4:**
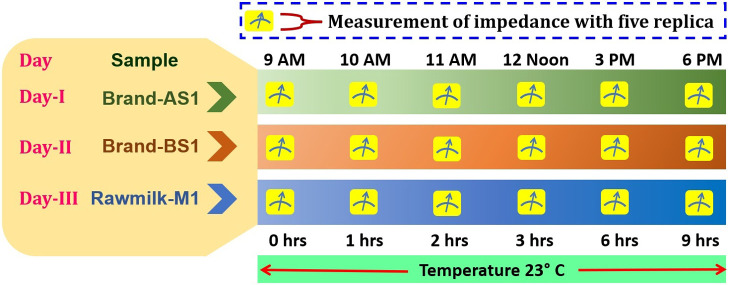
Measurement matrix of impedance for deterioration study of milk.

Next, the deterioration of milk at control temperature of 23°C was studied in terms of Cole parameters. The samples: Brand-AS1, Brand-BS1, Rawmilk-M1 were considered in this adulteration study. Impedances of these samples were measured and Cole parameters were extracted. In particular, these three samples were studied in three different days (Say Day-I, Day-II and Day-III respectively). Each day a single sample was investigated (between 9 AM to 6 PM). Samples were collected on their respective days of the experiment. At the beginning, the impedance was measured in 1 hour of interval. Once, significant difference in relaxation-time was found, the impedance was measured in 3 hours of interval. The measurement matrix for this investigation is depicted in Figure 4. Care was taken to minimize external factors like temperature and humidity that could affect the milk’s electrical properties. Care was also taken to minimize experimental errors, like human error, cleaning of sample holder, calibration of LCR meter etc.

## Results and discussion

In the first phase of the experiment, whether ‘Source’ of milk is factor or not was tested. In order to perform this test, impedances of all six samples (depicted in Table 1) were measured and respective relaxation-time were estimated. Based on the relaxation-time, ANOVA tests were performed and results of this study are tabulated in Table 2.

**Figure 5:**
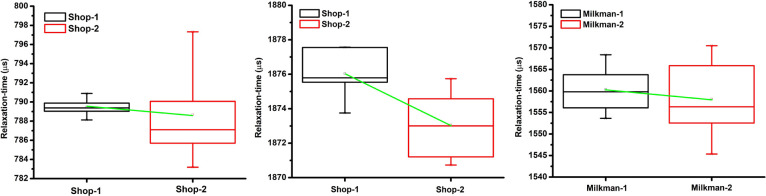
Variation relaxation-time with different sources of milk (left: Brand-A, middle: Brand-B, right: Raw milk). Statistically, it is found that ‘Source’ of a sample (i.e. Shop or Milkman) is not a factor, but brand is a factor.

The ANOVA test results indicate that there is no statistically significant variation in the relaxation-time among the milk samples taken from different shops (p>0.05) with 99% confidence. The results of this study indicate that the relaxation-time, a critical rheological parameter, did not show any statistically significant variation among the milk samples of a particular brand purchased from different shops. This implies that the processing, storage, and handling practices employed by the two retailers did not significantly impact the biophysical properties of milk. Therefore, consumers can have the confidence that the same brand of milk will have consistent rheological characteristics, regardless of the shop from which it is purchased. Technically, for raw milk too, the ‘Source’ is not a factor. Note the moderate p-value (0.085595) in case of raw milk (see Table 2). It could be due to the different manual processing by the milkmen. The variation of relaxation-time for different samples are shown in Figure 5. In addition to this, when ANOVA was performed for different brands, a clear distinguishability among the brands of the samples was noticed. In short, ‘Source’ is not a factor but ‘Brand’ is a factor (see Table 2). ‘Brand’ becomes a factor because of the following four main reasons: the difference in raw milk collection process adapted by the different brands, the differences in their posturizing process, the contamination level of different brands, the storage and handing process adapted by the different brands.

In the second phase of the experiment, time of adulteration of each brand was tested. In this course of experiment, a particular type of sample was investigated in a day and ‘time’ was considered as factor (see Figure 4 for sample test matrix). The samples were kept at control temperature and impedance was measured at every one-hour interval. Once significant difference in relaxation-time was observed, samples were tested at every three-hour interval. From the bioimpedance measurements Cole parameters were extracted and relaxation-time was estimated. Then ANOVA was performed based on relaxation-time obtained at different time intervals.

**Figure 6:**
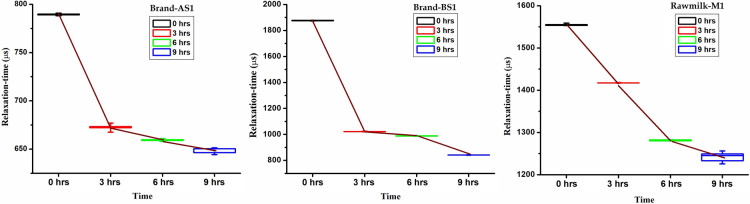
Variation of relaxation-time with adulteration time.

The raw data (i.e. Cole plots) can provide qualitative information and has been presented later in this section. No significant changes were observed by naked eye; neither any unusual smell was detected during the initial three hours of the adulteration process. The variation of relaxation-time and ANOVA test results are shown in Figure 6 and Table 3, respectively. From Figure 6, it is noticed that the relaxation-time decreases as adulteration progresses.

**Figure 7:**
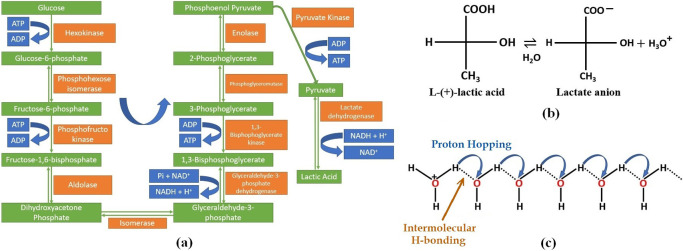
(a) Biochemical transformation of glucose into lactic acid in bacteria. (b) Dissociation of lactic acid. (c) H^+^ ionic conduction by Grotthus Mechanism.

Adulteration of milk is a complex biochemical transformation [26]. In case of raw milk, the Lactic Acid Bacteria (LAB), e.g., Lactobacilli, Lactococcus, etc., present naturally convert the principally present milk sugar, lactose which is a disaccharide of galactose and glucose into lactic acid. This process occurs through anaerobic oxidation of glucose and galactose (produced after hydrolysis of lactose in bacterial cytoplasm), i.e., glycolysis which produces pyruvic acid, followed by fermentation (specifically, lactic acid fermentation) with the help of the enzyme lactate dehydrogenase in most cases. This entire biochemical pathway is presented in Figure 7(a). In case of pasteurized milk when exposed to air, the anaerobic bacteria present in the air, contaminate the milk, and follow a similar biochemical pathway as described previously. Due to the presence of sugars, the bacterial growth is accelerated in the medium.

The lactic acid so produced will undergo dissociation, to produce a proton and a lactate anion (see Figure 7(b)). The H^+^ ion produced, increases the electrolytic conductivity due to its great electrical mobility and this can be explained by Grotthus Mechanism (see Figure 7(c)). This explains the decrease in relaxation-time with time.

The relaxation-time is directly related to rheological properties of milk. From the nature of the variation of relaxation-time in the first 3 hours, it can be noticed that the change is rapid for pasteurized milk in comparison to raw milk. Further, the values of relaxation-time for the samples Brand-BS1 and Rawmilk-M1 are comparable, indicating that the degree of contamination in these samples were practically the same. On the other hand, the degree of contamination in sample Brand-AS1 is significantly different as compared to other two samples.

**Figure 8:**
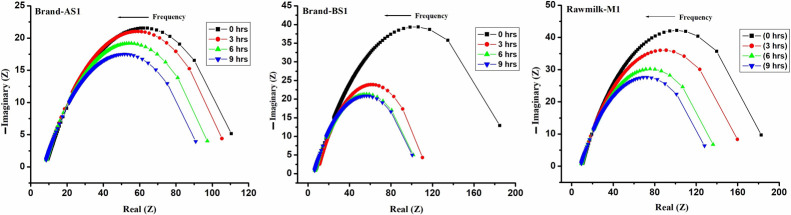
Cole plots for adulteration process. The unit of Z is Ω.

To describe the adulteration process Cole plots are drawn and are shown in Figure 8. From these Cole plots, the following interesting observations are noted. (a) for the all the samples, the order of real (as well as imaginary) impedances is same, even though their relaxation-time(s) are significantly different. (b) the range of real part at which the maxima of imaginary part occur are found to be in the range of 50 to 100 Ω. This could be due to the intrinsic electrical properties of milk. (c) As adulteration time progresses, the maxima of imaginary part shift towards the lower range of the real part.

During the adulteration process, the Cole parameters of the samples were extracted as described earlier. The extracted Cole parameters are tabulated in Table 4. It is interesting to note that the α parameter increases monotonically during the adulteration process for the pasteurized milk (Brand–AS1 and Brand–BS1), while the same monotonically decreases for the raw milk. To explain this fact, additional pathological tests are required (and it is beyond the scope of this work). In addition to this, the temperature dependence of deterioration is not studied here.

## Conclusion

Adulteration of milk is studied by means of bioimpedance spectroscopy, using Cole equivalent circuit with statistical relevance. A unique, low-cost sample holder was fabricated for studying electrical impedance of liquid sample. An experimental setup has been presented to estimate all Cole parameters using milk as liquid sample. It is found that the relaxation-time decreases as adulteration progresses for both branded milk as well as raw milk. The decrease in relaxation-time is found to be rapid in the first three hours of the adulteration process. The decrease in relaxation-time has been explained by the biochemical pathways involved. All kinds of liquid milk are found to deteriorate significantly within the first three hours and hence it is not advisable to consume milk, if kept at 23°C for two hours or more. It is possible to standardize our method as a cost-effective alternative to the expensive, conventional pathological tests in the monitoring of adulteration of liquid milk.

## Conflict of interest

The authors declare no conflict of interest related to this article.

## Author contributions

First author: Major role in the execution of experiments, data collection, data fitting, performing ANOVA. Second author: Fabrication of sample holder, sample collection, execution of experiments, performing ANOVA. Third author: Theoretical analyses and investigation on biochemical pathways; preparation of figures and images. Fourth author: Generated the research idea, proposed the research plan and supervised the work. All authors are equally participated in documentation.

## Data availability

The evidence data generated or analyzed during the current study are available from the corresponding author on reasonable request.

## Figures and Tables

**Table 1:** Sample matrix

Sample Name	Nature	Type	Source
Brand-AS1	Pasteurized	Brand-A	Shop-1
Brand-AS2	Pasteurized	Brand-A	Shop-2
Brand-BS2	Pasteurized	Brand-B	Shop-2
Rawmilk-M1	Raw milk	Non branded	Milkman-1
Rawmilk-M2	Raw milk	Non branded	Milkman-2

**Table 2:** ANOVA test result to check whether the ‘Source’ is a factor or not

Sample	Factor	p-value	F_value_	F_critical_	Distinguishability	Level of confidence
Brand-A	Different shop	0.758904	0.100883	11.25862	No	99%
Brand-B	Different shop	0.330052	1.075411	11.25862	No	99%
Rawmilk	Different Milkman	0.085595	3.843455	11.25862	No	99%

**Table 3:** ANOVA test result for ‘time’ adulteration of milk

Sample	Factor (Different time)	p-value	F_value_	F_critical_	Distinguishability	Level of confidence
Brand–A Day–I	9 AM and 12 noon	1.15E-12	5575.817	11.25862	Yes	99%
	9 AM and 3 PM	4.53E-16	39656.64	11.25862	Yes	99%
	9 AM and 6 PM	1.2E-13	9826.509	11.25862	Yes	99%
Brand–B Day–II	9 AM and 12 noon	4.82E-21	694210.9	11.25862	Yes	99%
	9 AM and 3 PM	2.13E-21	851653.3	11.25862	Yes	99%
	9 AM and 6 PM	3.72E-22	1317011	11.25862	Yes	99%
Rawmilk Day–III	9 AM and 12 noon	1.12E-14	17777.84	11.25862	Yes	99%
	9 AM and 3 PM	1.18E-16	55454.56	11.25862	Yes	99%
	9 AM and 6 PM	1.19E-11	3104.633	11.25862	Yes	99%

**Table 4:** Cole parameters during adulteration process

Sample	Time	R_0_ (Ω)	R∞ (Ω)	C (µF)	α	τ (µs)
Brand–AS1	0 hrs	116.6	8.2	295	0.48212	792
	3 hrs	100.5	7.5	304	0.48763	667
	6 hrs	113.3	7.8	244	0.49981	661
	9 hrs	107.6	7.5	258	0.49774	645
Brand–BS1	0 hrs	200.1	6.8	234	0.49287	1871
	3 hrs	106.9	5.7	289	0.51293	1022
	6 hrs	111.9	6.1	252	0.52361	986
	9 hrs	107.0	5.9	232	0.52998	841
Rawmilk–M1	0 hrs	192.0	10.0	154	0.55276	1554
	3 hrs	168.0	9.0	179	0.54255	1416
	6 hrs	143.0	8.7	206	0.53861	1280
	9 hrs	140.0	8.5	220	0.52842	1225
